# Patients' Experiences of Healthcare Professionals' Competence in Digital Counselling in Healthcare Settings—A Qualitative Systematic Review

**DOI:** 10.1111/jan.16663

**Published:** 2024-12-09

**Authors:** Juulia Kaihlaniemi, Petra Suonnansalo, Maria Kääriäinen, Pirjo Kaakinen, Maija Litendahl, Leila Paukkonen, Kirsi Laukkonen, Anne Oikarinen

**Affiliations:** ^1^ Research Unit of Health Sciences and Technology, Faculty of Medicine University of Oulu Oulu Finland; ^2^ The Finnish Centre for Evidence‐Based Health Care: A Joanna Briggs Institute Centre of Excellence Group Helsinki Finland; ^3^ MRC Oulu, Oulu University Hospital and University of Oulu Oulu Finland; ^4^ Wellbeing Services County of North Ostrobothnia Oulu Finland; ^5^ Diaconia University of Applied Sciences Oulu Finland; ^6^ Research Unit of Health Sciences and Technology, Faculty of Medicine, Medical Research Centre Medical Research Center Oulu, Oulu University Oulu Finland

**Keywords:** competence, digital counselling, experience, healthcare professionals, meta‐aggregation, patient, qualitative literature review, telenursing

## Abstract

**Aim:**

To critically appraise and synthesise qualitative evidence about patients' experiences of healthcare professionals' competence in digital counselling in healthcare settings.

**Design:**

A qualitative systematic review.

**Methods:**

The review followed the Joanna Briggs Institute methodology for systematic reviews of qualitative evidence. The review included studies that focused on patient experiences of healthcare professionals' competence in digital counselling and were published in English, Finnish or Swedish, with no time limits. Study selection, quality appraisal and data extraction were performed by two independent reviewers. Findings from the studies included were pooled using the meta‐aggregation method.

**Data Sources:**

Eight databases (Web of Science, CINAHL, Scopus, PsycArticles, Medic, Medline (Ovid), EBSCO Open Dissertations and MedNar) were systematically searched on 25 September 2023.

**Results:**

Sixteen studies (published between 2009 and 2023) were included in the review, from which 42 findings were extracted and organised into eight categories based on their meaning. Three synthesised findings were identified: (1) Competence to provide efficient digital counselling, (2) competence to support patient self‐management during digital counselling and (3) competence in establishing a reciprocal relationship in digital counselling.

**Conclusion:**

The evidence from the review can be used to support healthcare professionals' competence in digital counselling. It was found that competence in digital counselling includes the competence to provide digital counselling that is efficient and enables counselling to be implemented in health care, supports patients to self‐manage and establishes a reciprocal counselling relationship in a digital environment.

**Implications for the Profession and Patient Care:**

Recognising healthcare professionals' competence in digital counselling can enhance their motivation and professional growth, potentially improving the quality of services and patient outcomes. These findings can inform the development of healthcare education, fostering the training of more competent healthcare professionals and digital counsellors.

**Reporting Method:**

The review was undertaken and reported using the PRISMA guidelines.

**Protocol Registration:**

PROSPERO CRD42024499509.

No Patient or Public Contribution.

**Summary**

- What Does This Paper Contribute to the Wider Global Clinical Community?
  ○ Healthcare professionals may need to develop new competencies as counselling is increasingly moving to digital environments
  ○ Health care professionals need competence in providing efficient digital counselling, supporting patient self‐management during digital counselling and establishing a reciprocal relationship in digital counselling.
  ○ When educating and training healthcare professionals, it is crucial to address different aspects of digital counselling competence to ensure that they attain optimal expertise, thereby benefiting patients' health.

## 1 Introduction

With the emerging digitalisation of modern societies worldwide, digital technologies have become a regular practice within the healthcare sector (WHO 2020). Consequently, patient counselling increasingly takes place in digital environments (Koivunen and Saranto 2018; Paalimäki‐Paakki et al. 2022). Digital counselling enables real‐time connection between patients and healthcare professionals (HCPs), regardless of geographical distance (Bokolo 2020). Further, patients can prepare for appointments or procedures by reviewing instructions in advance from the comfort of their own homes (Kujala et al. 2020; Paalimäki‐Paakki et al. 2022). Digital counselling changes how HCPs work and new counselling competencies are, therefore, required (Paalimäki‐Paakki et al. 2022). Working in a digital counselling environment challenges the traditional counselling competence of HCPs who have previously self‐reported a lack of competence in areas such as communication in a digital environment (Laukka et al. 2020). HCPs must be able to meet patients' expectations of digital counselling and provide effective counselling in a digital environment, for example, interacting effectively (Powell, Stone, and Hollander 2018) and being comfortable with the digital platform on which the counselling takes place (Bokolo 2020).

The worldwide evolution of digital services has created new kinds of processes and infrastructures for delivering healthcare, reshaping the traditional characteristics of patient care (van Gemert‐Pijnen et al. 2011). Digital services enable interaction between HCPs and patients. They create further possibilities for sharing information at the right time during different stages of care (Oikarinen et al. 2023). It has been suggested that patients are satisfied (Gordon et al. 2020; Muñoz‐Tomás et al. 2023; Polinski et al. 2016), or even more satisfied (Polinski et al. 2016), with digital counselling than with traditional counselling, and that they see digital counselling as a preferred form of health appointment (Polinski et al. 2016). In the past, patient satisfaction has been described in terms of better access to care, shorter waiting times (Gordon et al. 2020; Powell, Stone, and Hollander 2018) and less travel‐related inconvenience (Orlando, Beard, and Saravana 2019). However, as research on digital counselling increases, patient satisfaction is no longer limited to these factors but relies increasingly on HCPs' competence.

Digital counselling at its best is patient‐centred, interactive, carefully planned and implemented with adequate resources (Kaakinen et al. 2020; Oikarinen et al. 2023). Interaction is an essential part of counselling, as a successful interactive relationship is crucial to enabling patients to take appropriate action and achieve their goals (Kaakinen et al. 2020; Oikarinen et al. 2018). Digital counselling draws on patients' knowledge as well as their psychosocial and physical needs (Kaakinen et al. 2020). Digital counselling environments facilitate the use of questionnaires and symptom diaries to enhance counselling and interaction between patients and HCPs (Kujala et al. 2020). When counselling is conducted in digital environments, the interaction between HCPs and patients changes. In the present study, digital counselling specifically refers to counselling that is conducted either by text‐based (Helzlsouer et al. 2016) or video‐mediated means (Buvik et al. 2016; Helzlsouer et al. 2016). Bokolo (2020) describes real‐time communication between a patient and HCP, for example, via video, as synchronous communication, and non‐time‐dependent communication, for example, via a health app, as asynchronous communication. Digital counselling can be provided by a wide range of HCPs, including nurses, physiotherapists and physicians.

Competence is a complex concept which is usually defined as a range of mutually supportive knowledge, skills, attitudes and values (Cowan, Norman, and Coopamah 2005; Mikkonen et al. 2018). In healthcare, practical (Cowan, Norman, and Coopamah 2005) and counselling skills are also emphasised. The literature identifies a range of competencies that HCPs need when counselling patients digitally, including digital competence (Jarva et al. 2022; Konttila et al. 2019; Kaihlaniemi et al. 2023), which may improve patient safety (Jarva et al. 2022). Previous studies have suggested that digital counselling competence comprises competence in providing patient‐centred care (Jarva et al. 2022) and supporting patients' self‐care, ethical competence, change competence and competence in creating an interactive counselling relationship and developing services (Kaihlaniemi et al. 2023). Digital services have created new challenges for HCPs, and a lack of experience (Bokolo 2020) and competence (Jarva et al. 2022) have affected their work in practice. It has been noted that HCPs might lack the competence to motivate and advise patients in self‐management (Kujala et al. 2020) or to communicate through patient portals (Laukka et al. 2020). Patient‐friendly designs are a starting point for the efficient use of digital solutions.

From the patients' point of view, it has been found that the biggest challenge in digital counselling is the way that HCPs interact (Powell, Stone, and Hollander 2018) and create a trusting relationship (Gordon et al. 2020) with them. The importance of interaction skills and creating an interactive counselling relationship has also been highlighted by HCPs themselves (Kaihlaniemi et al. 2023). Polinski et al. (2016) reported that the quality of care received was seen as a predictor of liking digital services. In general, digital services have been demonstrated to be effective, for example, in terms of self‐management (Zhu, Wong, and Wu 2018), and the patients who are satisfied with counselling have been found to be more committed to self‐management and to have better treatment outcomes (Oikarinen et al. 2018). Nevertheless, it is important to recognise vulnerable patient groups who may face challenges in using digital services. This will help to prevent the potential widening of health inequalities (Härkönen et al. 2024). It is important to explore patient experiences of HCPs' counselling competence, as patient expectations have been observed to facilitate adherence to digital interventions (Mohr, Cuijpers, and Lehman 2011).

Previous reviews have addressed digital services, including digital counselling, from various perspectives. An extensive umbrella review evaluated the impact of digital services on population health, service costs and patient and healthcare professional satisfaction, and identified the facilitators and barriers to using digital services in healthcare and social welfare (Härkönen et al. 2024). Other literature reviews have evaluated the effectiveness of digital counselling for different patient groups, such as heart failure (Allida et al. 2020; Zhang et al. 2024), knee osteoarthritis (Xie et al. 2021) and chronically ill patients (Paalimäki‐Paakki et al. 2022). A qualitative review considering HCPs' digital competence has also been published (Konttila et al. 2019). However, it seems that evidence about the patient experiences of HCPs' digital counselling competence is fragmented and there are no available reviews of this phenomenon.

## 2 The Review

### 2.1 Aim

To describe patient experiences of HCPs' competence in digital counselling in healthcare settings. The research question was: What experiences do patients have of professionals' competence in digital counselling in healthcare settings?

## 3 Methods

### 3.1 Design

This qualitative systematic review with meta‐aggregation was conducted according to the Joanna Briggs Institute guidelines for systematic reviews of qualitative evidence (Lockwood et al. 2020). It aimed to gather all the best evidence that describes adult patients' experiences of HCPs' competence in digital counselling in healthcare settings. The prior previously published PROSPERO protocol (CRD42024499509) guided the review process. The Preferred Reporting Items for Systematic Reviews and Meta‐Analyses (PRISMA) statement was applied when reporting the process of selecting the articles (Page et al. 2021, Supplementary File 1).

### 3.2 Search Methods

The PICo strategy for systematic reviews of qualitative evidence (Lockwood et al. 2020) was utilised, where the participants (P) were adult patients who had received digital counselling from a healthcare professional (HCP) in a healthcare setting, either for matters related to their own health or on behalf of someone else. The phenomenon of interest (I) was adult patients' experiences of HCPs' digital counselling competence, and the context (Co) was any healthcare setting where digital counselling was implemented. This review considered studies focusing on qualitative data.

The database search was performed on 25 September 2023, by an information specialist, and encompassed eight databases (Web of Science, CINAHL, Scopus, PsycArticles, Medic, Medline (Ovid), EBSCO Open Dissertations and MedNar). Before conducting the final database search, to develop the most suitable search strategy, various search terms and combinations of terms were tested. The search covered both published and unpublished studies. Therefore, grey literature (comprising unpublished studies) was searched for in the MedNar and Ebsco Open Dissertations databases. No date limitations were set, and the search was limited to studies published in Finnish, Swedish and English. The search strategies by the database are presented in Table 1.

**TABLE 1:** The search strategies by the database.

Search terms	Results retrieved
**CINAHL**	#4538
((MH "patient attitudes+") OR (MH "Patient Satisfaction") OR (MH "Life Experiences") OR ((Patient* OR outpatient* OR inpatient* OR customer* OR client* OR consumer*) N3 (experienc* OR perspectiv* OR perception* OR satisf* OR view* OR opinion* OR attitud*))) AND (((MH "patient education+") OR (MH "counseling+")) OR (”patient education” OR counsel* OR guidance OR ”informational support” OR ”informational knowledge” OR (information N2 giv*) OR (information N2 need*) OR coach*))) AND (“Remote counsel?ing” OR “Digital counsel?ing” OR “Distance counsel?ing” OR e‐therap* OR e‐counse?ling OR Telecare OR Teleconsultation OR Telecounse?ling OR “Text‐based counse?ling” OR “Audiovisual counse?ling” OR “Video counse?ling” OR Videoconferenc* OR “Video‐mediated interaction*” OR “Digital environment*” OR “Digital counsel?ing environment*” OR (MH "Videoconferencing+") OR (MH "Instant Messaging") OR (MH "Teleconferencing") OR (MH "Telehealth+") OR (MH "Internet‐Based Intervention") OR (MH "Remote Consultation") OR (MH "Cellular Phone+") OR (MH "Mobile Applications") OR (MH "Augmented Reality") OR (MH "Virtual Reality+") OR (MH "Information Technology+") OR (MH "Medical Informatics") OR (MH "Computing Methodologies+")) OR "Wearable Electronic Device*" OR telemedicine OR telehealth OR "Medical Informatics Application*" OR digital OR technolog* OR mobile OR online OR application* OR "information system*" OR "information network*" OR virtual OR internet OR "m‐health" OR "e‐health" OR mhealth OR ehealth OR smartphone* OR "Wearable Device*" OR smartwatch* OR chat OR "cell phone*" OR cellphone* OR tablet OR "connected health" OR "computer‐ and telephone‐delivered intervention*" OR "web‐based" OR ”augmented reality” OR ”mixed reality” OR ”360 VR”)	
**SCOPUS**	#9957
TITLE ABS KEY ((patient* OR outpatient* OR inpatient* OR customer* OR client* OR consumer*) W/3 (experienc* OR perception* OR perspectiv* OR satisf* OR view* OR opinion* OR attitud*)) AND TITLE‐ABS‐KEY ( "patient education" OR counsel* OR guidance OR "informational support" OR "informational knowledge" OR (information W/2 giv*) OR (information W/2 need*) OR coach*) AND TITLE‐ABS‐KEY ("remote counsel?ing" OR "distance counsel?ing" OR e‐therap* OR e‐counse?ling OR telecare OR teleconsultation OR telecounse?ling OR "text‐based counse?ling" OR "audiovisual counse?ling" OR "video counse?ling" OR videoconferenc* OR "video‐mediated interaction*" OR "instant messag*" OR teleconferenc* OR "internet‐based intervention*" OR "remote consultation" OR "information technology+" OR "medical informatics" OR "computing methodologies+" OR "wearable electronic device*" OR telemedicine OR telehealth OR digital OR technolog* OR mobile OR online OR application* OR "information system*" OR "information network*" OR virtual OR internet OR "m‐health" OR "e‐health" OR mhealth OR ehealth OR smartphone* OR "wearable device*" OR smartwatch* OR chat OR "cell* phone*" OR cellphone* OR tablet OR "connected health" OR "computer‐ and telephone‐delivered intervention*" OR "web‐based" OR "augmented reality" OR "mixed reality" OR "360 vr")) AND (LIMIT‐TO (LANGUAGE, "english") OR LIMIT‐TO (LANGUAGE, "swedish"))	
**MEDLINE**	#6049
exp Patient Satisfaction/ or exp Consumer Behavior/ or exp Life Change Events/ or ((Patient* or outpatient* or inpatient* or customer* or client* or consumer*) adj4 (experienc* or perspectiv* or perception* or satisf* or view* or opinion* or attitud*)).ab,kf,ti. AND exp Patient Education as Topic/ or exp Counseling/ or (patient education or counsel* or guidance or informational support or informational knowledge or coach*).ab,kf,ti. or (information adj3 giv*).ab,kf,ti. or (information adj3 need*).ab,kf,ti. AND exp "Telemedicine"/ or exp "Videoconferencing"/ or exp "Cell Phone"/ or exp "Internet‐Based Intervention"/ or exp "Mobile Applications"/ or exp "Computing Methodologies"/ or exp "Augmented Reality"/ or exp "Virtual Reality"/ or exp "Information Technology"/ or exp "Medical Informatics"/ or exp "Wearable Electronic Devices"/ or (Wearable Electronic Device* or telemedicine or telehealth or Medical Informatics Application* or digital or technolog* or mobile or online or application* or information system* or information network* or telephone‐delivered intervention* or virtual or internet or m‐health or e‐health or mhealth or ehealth or smartphone* or Wearable Device* or smartwatch* or chat or cell phone* or cellphone* or tablet or connected health or web‐based or augmented reality or mixed reality or 360 VR or Remote counsel?ing OR Digital counsel?ing OR Distance counsel?ing OR e‐therap* OR e‐counse?ling OR Telecare OR Teleconsultation OR Telecounse?ling OR Text‐based counse?ling OR Audiovisual counse?ling OR Video counse?ling OR Videoconferenc* OR Video‐mediated interaction* OR Digital environment* OR Digital counsel?ing environment*).ab,kf,ti.	
**Ebsco open dissertations**	#275
( ( patient* OR outpatient* OR inpatient* OR customer* OR client* OR consumer* ) N3 ( experienc* OR perception* OR perspectiv* OR satisf* OR view* OR opinion* OR attitud* ) ) AND ( "patient education" OR counsel* OR guidance OR "informational support" OR "informational knowledge" OR coach* OR ( information N2 giv* ) OR ( information N2 need* ) ) AND ( "remote counsel?ing" OR "distance counsel?ing" OR e‐therap* OR e‐counse?ling OR telecare OR teleconsultation OR telecounse?ling OR "text‐based counse?ling" OR "audiovisual counse?ling" OR "video counse?ling" OR videoconferenc* OR "video‐mediated interaction*" OR "instant messag*" OR teleconferenc* OR "internet‐based intervention*" OR "remote consultation" OR "information technology+" OR "medical informatics" OR "computing methodologies+" OR "wearable electronic device*" OR telemedicine OR telehealth OR digital OR technolog* OR mobile OR online OR application* OR "information system*" OR "information network*" OR virtual OR internet OR "m‐health" OR "e‐health" OR mhealth OR ehealth OR smartphone* OR "wearable device*" OR smartwatch* OR chat OR "cell* phone*" OR cellphone* OR tablet OR "connected health" OR "computer‐ and telephone‐delivered intervention*" OR "web‐based" OR "augmented reality" OR "mixed reality" OR "360 vr" )	
**Mednar**	#359
( ( patient* OR outpatient* OR inpatient* OR customer* OR client* OR consumer* ) AND ( experienc* OR perception* OR perspectiv* OR satisf* OR view* OR opinion* OR attitud* ) ) AND ( "patient education" OR counsel* OR guidance OR "informational support" OR "informational knowledge" OR coach* OR ( information AND giv* ) OR ( information AND need* ) ) AND ( "remote counsel?ing" OR "distance counsel?ing" OR e‐therap* OR e‐counse?ling OR telecare OR teleconsultation OR telecounse?ling OR "text‐based counse?ling" OR "audiovisual counse?ling" OR "video counse?ling" OR videoconferenc* OR "video‐mediated interaction*" OR "instant messag*" OR teleconferenc* OR "internet‐based intervention*" OR "remote consultation" OR "information technology+" OR "medical informatics" OR "computing methodologies+" OR "wearable electronic device*" OR telemedicine OR telehealth OR digital OR technolog* OR mobile OR online OR application* OR "information system*" OR "information network*" OR virtual OR internet OR "m‐health" OR "e‐health" OR mhealth OR ehealth OR smartphone* OR "wearable device*" OR smartwatch* OR chat OR "cell* phone*" OR cellphone* OR tablet OR "connected health" OR "computer‐ and telephone‐delivered intervention*" OR "web‐based" OR "augmented reality" OR "mixed reality" OR "360 vr" )	
**PsykArticles**	#148
( ( patient* OR outpatient* OR inpatient* OR customer* OR client* OR consumer* ) N3 ( experienc* OR perception* OR perspectiv* OR satisf* OR view* OR opinion* OR attitud* ) ) AND ( "patient education" OR counsel* OR guidance OR "informational support" OR "informational knowledge" OR coach* OR ( information N2 giv* ) OR ( information N2 need* ) ) AND ( "remote counsel?ing" OR "distance counsel?ing" OR e‐therap* OR e‐counse?ling OR telecare OR teleconsultation OR telecounse?ling OR "text‐based counse?ling" OR "audiovisual counse?ling" OR "video counse?ling" OR videoconferenc* OR "video‐mediated interaction*" OR "instant messag*" OR teleconferenc* OR "internet‐based intervention*" OR "remote consultation" OR "information technology+" OR "medical informatics" OR "computing methodologies+" OR "wearable electronic device*" OR telemedicine OR telehealth OR digital OR technolog* OR mobile OR online OR application* OR "information system*" OR "information network*" OR virtual OR internet OR "m‐health" OR "e‐health" OR mhealth OR ehealth OR smartphone* OR "wearable device*" OR smartwatch* OR chat OR "cell* phone*" OR cellphone* OR tablet OR "connected health" OR"computer‐ and telephone‐delivered intervention*" OR "web‐based" OR "augmented reality" OR "mixed reality" OR "360 vr" )	
**Web of science**	# 4060
( patient* OR outpatient* OR inpatient* OR customer* OR client* OR consumer* ) NEAR/3 ( experienc* OR perception* OR perspectiv* OR satisf* OR view* OR opinion* OR attitud* ) (Topic) and "patient education" OR counsel* OR guidance OR "informational support" OR "informational knowledge" OR coach* OR ( information NEAR/2 giv* ) OR ( information NEAR/2 need* ) (Topic) and remote counsel?ing" OR "distance counsel?ing" OR e‐therap* OR e‐counse?ling OR telecare OR teleconsultation OR telecounse?ling OR "text‐based counse?ling" OR "audiovisual counse?ling" OR "video counse?ling" OR videoconferenc* OR "video‐mediated interaction*" OR "instant messag*" OR teleconferenc* OR "internet‐based intervention*" OR "remote consultation" OR "information technology+" OR "medical informatics" OR "computing methodologies+" OR "wearable electronic device*" OR telemedicine OR telehealth OR digital OR technolog* OR mobile OR online OR application* OR "information system*" OR "information network*" OR virtual OR internet OR "m‐health" OR "e‐health" OR mhealth OR ehealth OR smartphone* OR "wearable device*" OR smartwatch* OR chat OR "cell* phone*" OR cellphone* OR tablet OR "connected health" OR "computer‐ and telephone‐delivered intervention*" OR "web‐based" OR "augmented reality" OR "mixed reality" OR "360 vr" (Topic)	
**Medic** (Finnish database)	#89
digi* etä* AND ohja* potilasohja* neuvon*	

### 3.3 Inclusion and Exclusion Criteria

Study selection was based on specific inclusion and exclusion criteria that were, in turn, based on criteria developed according to the PICo protocol as follows: (1) the study focuses on patients over 18 years of age; (2) the patients had received digital counselling for themselves or on behalf of someone else; (3) the study considers patients' views and experiences of HCPs' digital counselling competence (knowledge, skills or attitudes), (4) within which communication between patients and HCPs was reciprocal (asynchronous or synchronous), that is, the counselling was implemented through text‐based and/or video‐mediated methods, (5) and in which digital counselling was provided by HCPs with various job titles, including but not limited to registered nurses, speech therapists, physiotherapists, occupational therapists, psychologists and physicians and (6) qualitative studies including, but not limited to, designs such as phenomenology, qualitative description, action research, grounded theory, ethnography, feminist research and qualitative results from mixed‐method studies.

The exclusion criteria were as follows: (1) patients had received counselling via text message, phone, social media (e.g., WhatsApp, Facebook) or email, (2) no patient–HCP interaction or the interaction was one‐sided (e.g., the patient simply viewed digital material, filled in health‐related questionnaires or used chatbots).

### 3.4 Study Selection

All citations that were identified were uploaded into Covidence, and duplicates were eliminated. The studies that were identified underwent eligibility screening based on their title and abstract, followed by full‐text screening by two independent reviewers (two of JK, PS, PK, ML, LP, KL and AO) using the predefined inclusion and exclusion criteria. Any differences of opinion between the reviewers were resolved through discussion or with the involvement of a third reviewer.

### 3.5 Search Outcome

**FIGURE 1:**
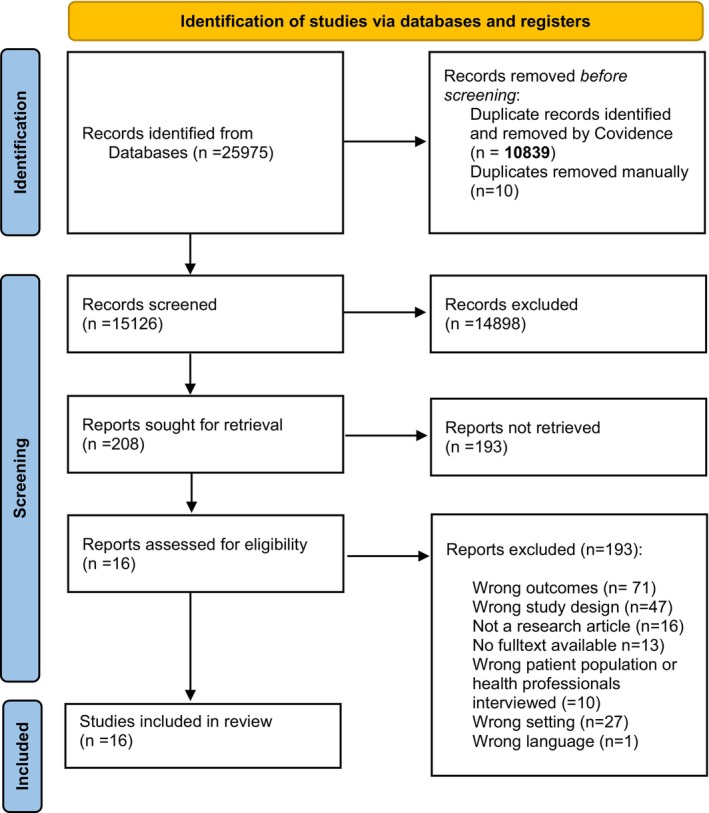
PRISMA flow diagram for search results and study selection and inclusion process (Page et al. 2021).

Altogether, the database search identified 25,975 articles. All data were uploaded to Covidence which identified duplicates (*n* = 10,849), after which 15,126 titles and abstracts were screened. In total, 14,898 articles were excluded in this phase, leaving 208 full texts of articles to be reviewed for eligibility. Of these full texts, 193 more were excluded because they did not meet the inclusion criteria (Figure 1), leaving 16 studies for critical appraisal.

### 3.6 Quality Appraisal

The JBI Critical Appraisal Checklist for Qualitative Research was used to assess the quality of the studies (Lockwood et al. 2020). The quality appraisal process was done in Covidence. All the studies selected for the review after full‐text evaluation were independently evaluated for methodological quality by two researchers (two of JK, PS, PK, ML, LP, KL and AO). The checklist comprises 10 items, each scored as ‘yes (=1)’, ‘no (=0)’ or ‘unclear’, with a maximum point allocation of 10. The reviewers resolved any disagreements through discussion or with the involvement of a third reviewer. As guided by the JBI protocol, no studies were excluded based on the quality appraisal.

### 3.7 Data Extraction and Synthesis

The data from the selected studies (*n* = 16) were extracted based on the specific details outlined in each study, utilising the standardised JBI data extraction tool, JBI SUMARI. The extracted details encompassed participants, study setting, geographical location, study methods, phenomena of interest, results and the type of digital counselling described in the study.

Two independent reviewers (JK and PS) pooled and synthesised the data using a meta‐aggregation approach as described by Lockwood et al. (2020). This method is used to compile findings and illustrations from qualitative studies, categorising them based on the similarity of meanings (Aromataris and Munn 2020). Findings were extracted according to the themes or subthemes presented in each article, taking into consideration whether they included illustrative quotations directly relating to patient experiences of HCPs' competence in digital counselling. Only credible findings (*C* = accompanied by an illustration lacking a clear association and therefore open to challenge) and unequivocal findings (*U* = accompanied by an illustration beyond reasonable doubt and not open to challenge) were included in the synthesis. In a synthesis like this, the reviewers can decide the level (themes or subthemes) that is most representative of the phenomenon of interest.

This procedure involved directly extracting categories as they appeared and subsequently combining or synthesising the results to formulate a series of statements that encapsulate the aggregated data. This was accomplished by organising and grouping the findings based on shared meanings. Each category was assigned a label reflecting its content, and these categories were then synthesised into a set of consolidated findings.

## 4 Results

### 4.1 Assessment of Methodological Quality of Included Studies

The 16 studies were reviewed for methodological quality, and all were found to be methodologically good (Table 2), the quality ranging from moderate to high as follows: three articles (Gilbert et al. 2022; O'Brien et al. 2023; Sayar, Vøllestad, and Nordgreen 2023) met all 10 of the critical appraisal criteria, two articles (Kringle et al. 2023; Remes, Hakala, and Oikarinen 2023) met nine criteria, six articles (Atkinson 2023; Danbjørg et al. 2015; Lindberg, Christensson, and Ohrling 2009; Marent et al. 2021; Nissen and Lindhardt 2017; Roslan et al. 2024) met eight of the criteria and five (Dubrofsky et al. 2023; Ehrenreich et al. 2019; Elliott et al. 2020; Rief et al. 2017; Zilliacus et al. 2010) met seven criteria. The evaluation criteria and assessment of the study articles are presented in Table 2.

**TABLE 2:** The evaluation criteria and assessment of methodological quality of included studies (*n* = 16).

Study	Q1	Q2	Q3	Q4	Q5	Q6	Q7	Q8	Q9	Q10	Total
Atkinson (2023)	Y	Y	Y	Y	Y	N	N	Y	Y	Y	8/10
Danbjørg et al. (2015)	Y	Y	Y	Y	Y	N	N	Y	Y	Y	8/10
Dubrofsky et al. (2023)	N	Y	Y	Y	Y	N	N	Y	Y	Y	7/10
Ehrenreich et al. (2019)	N	Y	Y	Y	Y	N	N	Y	Y	Y	7/10
Elliott et al. (2020)	Y	Y	Y	Y	Y	N	N	Y	N	Y	7/10
Gilbert et al. (2022)	Y	Y	Y	Y	Y	Y	Y	Y	Y	Y	10/10
Kringle et al. (2023)	Y	Y	Y	Y	Y	Y	N	Y	Y	Y	9/10
Lindberg, Christensson, and Ohrling (2009)	Y	Y	Y	Y	Y	N	N	Y	Y	Y	8/10
Marent et al. (2021)	Y	Y	Y	Y	Y	N	N	Y	Y	Y	8/10
Nissen and Lindhardt (2017)	Y	Y	Y	Y	Y	N	N	Y	Y	Y	8/10
O'Brien et al. (2023)	Y	Y	Y	Y	Y	Y	Y	Y	Y	Y	10/10
Remes, Hakala, and Oikarinen (2023)	N	Y	Y	Y	Y	Y	Y	Y	Y	Y	9/10
Rief et al. (2017)	N	Y	Y	Y	Y	N	N	Y	Y	Y	7/10
Roslan et al. (2024)	Y	Y	Y	Y	Y	N	N	Y	Y	Y	8/10
Sayar, Vøllestad, and Nordgreen (2023)	Y	Y	Y	Y	Y	Y	Y	Y	Y	Y	10/10
Zilliacus et al. (2010)	N	Y	Y	Y	Y	N	N	Y	Y	Y	7/10
%	31,3	100	100	100	100	68,8	75	100	6,3	100	—

*Note:* JBI evaluation criteria for qualitative research: Q1 = Is there congruity between the stated philosophical perspective and the research methodology. Q2 = Is there congruity between the research methodology and the research question or objectives? Q3 = Is there congruity between the research methodology and the methods used to collect data? Q4 = Is there congruity between the research methodology and the representation and analysis of data? Q5 = Is there congruity between the research methodology and the interpretation of result? Q6 = Is there a statement locating the researcher culturally or theoretically? Q7 = Is the influence of the researcher on the research and vice‐versa addressed? Q8 = Are participants and their voices adequately represented? Q9 = Is there research ethical according to current criteria or, for recent studies, and is there evidence of ethical approval by an appropriate body? Q10 = Do the conclusions drawn in the research report flow from the analysis, or interpretation of the data?

Abbreviations: N = no, U = unclear, Y = yes.

### 4.2 Overview of the Studies Reviewed

The characteristics of the studies included in the review are presented in Table 3. The original studies in the review were published between 2009 and 2023 across 10 countries: the USA (4), the United Kingdom (3), Denmark (2), Canada (1), Sweden (1), Ireland (1), Finland (1), Malaysia (1), Norway (1) and Australia (1).

**TABLE 3:** Characteristics of the studies included in the review (*n* = 16).

Study	Methods for data collection	Data analysis	Country	Phenomena of interest	Setting	Participant characteristics and sample size	Main results	The type of digital counselling
Atkinson (2023)	Qualitative interviews	Thematic analysis	UK	How alcohol treatment clients make sense of the relational aspects of therapy delivered remotely, and how the use of remote therapy might disrupt existing ideas around the therapeutic relationship	A single national drug and alcohol treatment service provider	15 adult service users	Four main themes emerged from the data: (1) The client in their own home, (2) Virtual relationships, (3) Empowerment and choice and (4) Alcohol—stigma and dependency.	Videoconferencing (Skype)
Danbjørg et al. (2015)	An interpretative perspective using qualitative methods A qualitative interview study	Systematic text condensation (STC) based on Giorgi's descriptive phenomenological method	Denmark	How postnatal parents experienced the use of telemedicine following early hospital discharge (i.e., 24 h after childbirth)	Postnatal ward	28 sets of parents, including 27 mothers and 11 fathers, were interviewed	Three categories were identified: (1) Timely information gives a feeling of control, support and reassurance, (2) Technology provides an accessible means of informing, supporting and guiding new parents and (3) Written asynchronous communication offers an accessible way to seek help after early discharge	An app with chat, knowledge base, and automated messaging features
Dubrofsky et al. (2023)	Telephone focus groups A qualitative descriptive study	Thematic analysis	Canada	Patient perspectives on a pilot virtual follow‐up programme after hypertensive disorders of pregnancy	Pilot educational website	16 participants with a hypertensive disorder of pregnancy (HDP) in the previous 5 years	Themes were separated into three categories: (1) patient experience prior to the programme, (2) feedback on the Her‐HEART programme and (3) patient perspective on long‐term follow‐up after experiencing an HDP	An online educational tool with virtual consultations by a nephrology/hypertension specialist
Ehrenreich et al. (2019)	In‐depth telephone interviews	An iterative thematic analysis	USA	To evaluate patients' experiences using telemedicine to attend abortion information visits	One PPAU clinic provided abortion care in Salt Lake City and seven PPAU‐affiliated clinics provided information visits but not abortion care	18 women who used telemedicine to attend state‐mandated information visits	The results describe women's experiences in terms of reasons for choosing telemedicine, cost and scheduling, privacy and experience using telemedicine	Videoconferencing
Elliott et al. (2020)	An appreciative inquiry approach Voluntarily submitted patient comments associated with a 5‐star review after a visit were randomly selected from more than 49,000 comments in an 11‐month period	A qualitative analysis of patient visit feedback	USA	Building consensus around exemplary interpersonal and communication practices during a virtual urgent care visit from the patients' point of view.	A proprietary platform	A total of 3560, 5‐star comments with corresponding positive reviews	A final set of codes included (1) Builds Rapport; (2) Patient Perspective; (3) Expectation and Agenda Setting; (4) Elicits Information; (5) Listens, Is Attentive; (6) Shares Information/Provides Guidance; (7) Shares Decision‐Making; (8) Spent Right Amount of Time; (9) User Experience; (10) Uncodable and (11) Provided Treatment	Proprietary platform enabling video visits on smartphones, tablets or computers
Gilbert et al. (2022)	Semistructured interviews conducted over the telephone or by video call	Abductive analysis	UK	To investigate the experiences of the patients, clinicians, and managers during the accelerated implementation of virtual consultations (both phone and video consultation) due to COVID‐19.	Specialist Orthopaedic Hospital in North London	20 patients with experience of orthopaedic/musculoskeletal condition and attending the research site for physiotherapy or occupational therapy	Key mechanisms that contribute to the formulation of patient preferences were identified. These were: (1) context for the consultation (normative expectations, relational expectations, congruence and potential), (2) the available alternatives and the implementation process (coherence, cognitive participation, collective action and reflexive monitoring)	Virtual consultation, a collective term for phone and video consultations
Kringle et al. (2023)	Semistructured interviews over the telephone within 2 weeks after the last group	Inductive thematic analysis	USA	Explore stakeholders' experiences with engaging in a group‐based intervention delivered through videoconferencing during the COVID‐19 pandemic and identify barriers, facilitators and strategies that may enable remote intervention delivery using videoconferencing among adults with stroke‐related disability	Community‐based telerehabilitation	8 low‐income adults with chronic stroke	Two overall themes were identified. (1) stakeholder experiences with the ENGAGE intervention during the COVID‐19 pandemic (2) stakeholder experiences using videoconferencing software to access and engage in intervention	Videoconferencing (Zoom)
Lindberg, Christensson, and Ohrling (2009)	Semistructured interviews 6 weeks after childbirth	A descriptive perspective using both quantitative and qualitative methods was used Thematic content analysis	Sweden	Parents' experiences of using videoconferencing when discharged early from a maternity unit	Maternity department and new parents in their home	18 parents (the interviews with the fathers and the mothers were initially analysed separately)	The analysis revealed four categories: (1) feeling confident with the technology, (2) feeling confident of having control of their privacy, (3) feeling confident being face‐to‐face on the videoconferencing, (4) feeling confident when worries and concerns were met, and answers were received	Follow‐up video communication with the midwife at the department, incorporating both audio and visual elements.
Marent et al. (2021)	Codesign workshops and interviews	Coding procedures in grounded theory	UK	The aim was to assess the implications of the digital care pathway by understanding patients' and healthcare professionals' experiences of the opportunities and restrictions—affordances—of different forms of doctor–patient interactions	Clinics in five European cities (Barcelona, Brighton, Lisbon, Zagreb and Antwerb) that participated in the EmERGE‐study	34 stable HIV patients, who have used EmERGE platform for more than 6 months.	Three themes: (1) Spatial dimension: from colocated bodies to informational scopes, (2) Temporal dimension: from synchronous copresence to response presence, (3) Social dimension: from diffuse to specific relationships	EmERGE‐platform (Digital Care Pathway): the doctor would send information and messages to the patient's smartphone app
Nissen and Lindhardt (2017)	Descriptive design using semistructured interviews	Manifest and latent content analysis	Denmark	Stable COPD patients' experiences of participating in a 6‐month telemedicine intervention substituting visits to the outpatient clinic	Four Danish hospitals participating in the Net‐COPD project	14 patients with stable COPD	Three themes emerged from the data: (1) a sense of security and control, (2) knowing your disease, (3) the virtues of virtual consultations	Video consultation, including measuring spirometry
O'Brien et al. (2023)	A qualitative descriptive study Critical realist philosophy Semistructured interviews	Thematic analysis	Ireland	Experiences of participants who engaged in a telehealth, multidisciplinary rehabilitation programme for UGI cancer survivors	The National Centre for Oesophago‐gastric Cancer in Ireland	10 participants	Three overarching themes: (1) ReStOre@Home impacted psychosocial and physical needs by addressing a broad and meaningful gap in services, (2) paving a pathway towards prosperity and (3) contrasting experiences with using technology	Digital platform offering remote group resistance training, monitored aerobic workouts, one‐to‐one dietetic counselling, individual support calls, and group education
Remes, Hakala, and Oikarinen (2023)	A descriptive qualitative approach with individual thematic interviews via Teams	Inductive content analysis	Finland	Endometriosis patients' experiences of the counselling they need from the nurses through the digital care pathway	A gynaecological outpatient clinic at a university hospital	14 women with endometriosis	Four main categories were revealed: (1) counselling on endometriosis and its role in life; (2) counselling on how to live with endometriosis; (3) comprehensive support for self‐care and (4) patient‐oriented counselling.	Digital CarePath
Rief et al. (2017)	A descriptive qualitative study Part of larger Randomised controlled trial	Thematic analysis	USA	Participant experiences and satisfaction with an active Personal Health Record (PHR)	Documents in the health care system of patients with cardiovascular diseases	41 participants in 5 focus groups	Three main themes emerged from the data (1) patient‐driven communication; (2) partnering with providers and (3) increasing awareness and proactivity in tracking	Active Personal Health Record
Roslan et al. (2024)	A qualitative study Semistructured group discussions and in‐depth individual interviews	Thematic analysis	Malaysia	Malaysian dental undergraduates' and patients' experiences undergoing smoking cessation counselling through virtual platforms	In dental care clinic	23 students (group interviews) and 9 patients (individual interviews)	Themes that emerged: general opinions and experiences, content of VCs, remote access to counselling, patient–clinician relationships, technical issues, changes after VCs and future application	Virtual platform
Sayar, Vøllestad, and Nordgreen (2023)	An online survey, with open‐ended questions	Conventional content analysis approach	Norway	The aim was to explore what patients missed in the contact with their therapist in guided Internet‐delivered cognitive behavioural therapy (ICBT) in routine care	A public secondary mental health care outpatient clinic in Bergen, Norway	579 patients with panic disorder, social anxiety disorder or major depressive disorder who received guided ICBT.	The analysis yielded three main categories: (1) therapist‐ascribed shortcomings; (2) programme obstacles and (3) self‐attributed limitations	Guided ICBT is a structured form of psychological treatment available online through a computer or mobile phone, with counselling provided
Zilliacus et al. (2010)	A qualitative study Semistructured telephone interviews	The conceptual framework of Miles and Huberman (1984) guided the analysis	Australia	Aims were to explore women's experiences with telemedicine, and their satisfaction with the technology and with the interactions with their genetic clinicians and counsellors, particularly in regard to their sense of social presence via videoconferencing	A Familial Cancer Service in New South Wales	Twelve women who had received telemedicine genetic counselling for hereditary breast and/or ovarian cancer (HBOC) within the previous 12 months	The results describe women's experiences in terms of overall experience and satisfaction, technical aspects, expectations and purpose of the consultation, social presence, interacting with the genetic clinician, interacting with the genetic counsellor, advantages of telegenetics, disadvantages and learning from one participant's negative experience	Videoconferencing systems

Across the selected studies, digital counselling was conducted via Skype (Atkinson 2023), an app with a chat, a knowledge base and automated messaging features (Danbjørg et al. 2015; Marent et al. 2021), an online educational tool with virtual consultations (Dubrofsky et al. 2023), video consultations (Ehrenreich et al. 2019; Kringle et al. 2023; Nissen and Lindhardt 2017; Zilliacus et al. 2010; Sayar, Vøllestad, and Nordgreen 2023; Lindberg, Christensson, and Ohrling 2009), a platform enabling video visits on smartphones, tablets or computers (Elliott et al. 2020), virtual consultations, encompassing both phone and video formats (Gilbert et al. 2022), a digital platform offering remote group resistance training, monitored aerobic workouts, one‐to‐one dietetic counselling, individual support calls and group education (O'Brien et al. 2023), Digital Care Path (Remes, Hakala, and Oikarinen 2023), Active Personal Health Record (Rief et al. 2017) and a virtual platform offering online therapy, including counselling, through a computer or mobile phone (Roslan et al. 2024).

### 4.3 Patient Experiences of HCPs' Competence in Digital Counselling in Healthcare Settings

From the studies included, 41 findings were identified that answered the research question. These were aggregated into 13 categories and then into three synthesised findings as follows: (1) competence to provide efficient digital counselling, (2) competence to support patient self‐management during digital counselling and (3) competence in establishing a reciprocal relationship in digital counselling. The findings, illustrations and respective levels of credibility are described in Table 4, and a summary of findings is presented in Table 5.

**TABLE 4:** Findings, illustrations and level of credibility.

Findings	Illustration	Level of credibility
Category 1: Competence in providing digital support to patients		
Zoom functions	Make sure that they were familiar with the particular interface on their device (G09, p.7) Kringle et al. (2023)	U
Checklist	You would have to set up like in a checklist to make sure everybody's comfortable you know, with all of the buttons and what it can do (G17, p.7) Kringle et al. (2023)	U
Demonstration	On the very first session… facilitators were able to share their screen to show someone how, where to click and you know, what to look for on their screen. And that was really helpful (Caregiver G18, p.7) Kringle et al. (2023)	U
Repetition	Let them play with it for a little while. So like me, I have a short memory. So you know, I can do it this week but next week I may forget (G05, p.7) Kringle et al. (2023)	U
Written materials	The Zoom paper that they give you at the start of the group study that (G15, p.7) Kringle et al. (2023)	U
Contrasting experiences with using technology	An education session on the watch should be included… Just how to use that, to give tips and tricks and that kind of stuff (P09, p.8) O'Brien et al. (2023)	U
Category 2: Competence in solving technological problems		
Technology disruptions	[The study staff] and the other facilitators involved would be available to walk them through and troubleshoot the issues fairly well and it didn't cause too much of a disruption (G09, p.6) Kringle et al. (2023)	U
Collective action	But it just kept cutting out, but I'm not sure whether that's her connection or whether it's my end connection. It was kind of annoying. But if cut out she'd phone me or, as I say earlier, we started doing (x software), and it sort of worked better and did the trick. So (x software) worked better (P4–16, p.783) Gilbert et al. (2022)	U
Category 3: Competence in making treatment‐related decisions		
Elicits information	She asked me a lot of questions to see exactly what was wrong with me (p.311) Elliott et al. (2020)	U
Shares decision making	Looked up the facts and made sure my concerns were taken care of. Also formed a plan of action with me (p. 312) Elliott et al. (2020)	U
Provides treatment	He diagnosed my sinus infection quickly and prescribed the medication I needed (p. 312) Elliott et al. (2020)	U
Category 4: Competence in providing instructions related to self‐management		
Written asynchronous communication offers an accessible way to seek help after early discharge	They were easy to understand and they were long, useful answers, not just short: you have to do this and that. There was an explanation; you have to do this and that, because of this (Mother 6, primipara, p.579) Danbjørg et al. (2015)	C
Shares information/Provides guidance	I liked how she explained everything and how she instructed me on how to test for various things, and how to keep monitoring him from home (p. 311) Elliott et al. (2020)	U
Pushing	He could've pushed me into doing several, and more challenging exposures. It is always easy to take the path of least resistance when things are difficult (p.5) Sayar, Vøllestad, and Nordgreen (2023)	U
Tailored contact	Tips for exposing, specific exercises and ways to narrow areas for exposure. It was difficult to judge whether I progressed too slowly (p.5) Sayar, Vøllestad, and Nordgreen (2023)	C
Personalised care meeting and supporting individual needs	I hurt my back just as the programme was getting going… The guys were able to help me with that…hey took some exercises o/ that would have been detrimental to the back, we did it that way, it was great (P09, p.7) O'Brien et al. (2023)	U
Feelings of connectedness with peers and professionals	I think [researcher)] has a lovely way about her. Engaging, pushing you on, and communicating, which, besides all her professional skills, I think that really helped. That's what I mean about the caring element of it (P11, p5) O'Brien et al. (2023)	C
Reflexive monitoring	I mean like correcting someone's movements you can do it over the video, but I'm not sure how accurate that it. It could be accurate if the video quality is good, but less maybe if it's not that great. What else? I'm not sure (P4–1, p.783) Gilbert et al. (2022)	U
Patient‐driven communication	My physician is great, she would always call me back—but it's really nice to know that I can just send her a quick email, you know: ‘this is where we are, you know, and we talk about this is what's happening, what should I do?’ You know, that is, it's almost like another level of comfort. She's just an email away (p 313) Rief et al. (2017)	U
Category 5: Competence in providing patients with information about their disease and its treatment		
Virtual consultation empowered self‐advocacy	I feel like it empowered me to know what follow‐up I should ask for in the future. And you know, we talked a little bit about testing blood pressure, and how often that should be done and simple things like that… So little things like that sort of helps [sic] with advocacy I guess going forward and knowing what the things are to watch for. I think I've never been very good at–like I've always been a little bit intimidated by doctors and that process. And so I think feeling like I just have a bit better understanding of my own health and things I should watch for going forward I think will make sure that I advocate a little bit better for myself (Group B, Participant 1, p.467) Dubrofsky et al. (2023)	U
Counselling on how to live with endometriosis	For such support or some kind of ticket tags that could be distributed to loved ones, and especially to the husband. Even though he says he understands, and will surely understand, it is hard for him to look at his wife crawling and vomiting; this takes great strength from him too (Participant 10, p.3) Remes, Hakala, and Oikarinen (2023)	U
Comprehensive support for self‐care	If necessary, it would be good to have an opportunity to speak with a nurse who has expertise in endometriosis. It cannot be assumed that all healthcare providers have knowledge of endometriosis, but at least one nurse with such expertise should always be available (Participant 3, p.3) Remes, Hakala, and Oikarinen (2023)	U
Counselling on endometriosis and its role in life	However, because it is a chronic disease, it can be associated with so much of the so‐called vagueness that you may not think of yourself or even be able to combine as symptoms until the diagnosis has been made …(−)… if we would get extensive information about the symptoms then we could connect things (Participant 5, p.3) Remes, Hakala, and Oikarinen (2023)	U
Improved physical and mental health	I think it might be worth explaining again what change has happened to your body in simple English… your stomach and your diaphragm… Maybe I'm wrong, I'm not so sure everybody fully understood that (P11, p.7) O'Brien et al. (2023)	C
Knowing your disease	I'm satisfied that there's more focus on it. I mean (…) that it gives this great sense of security…there's an eye on your disease (…) that you don't have to get hospitalised. That you get treated in time, instead of just waiting a week … (Participant 3, p.14) Nissen and Lindhardt (2017)	U
Category 6: Competence in building trusting relationship		
Partnering with providers	As a patient dealing with health care I feel… a need to be a part of that [the process], so I like that… it provides me with, you know, a check point… so I can be in partnership with, with my doctors. So I'm not relying on them to tell me when this is [occurring]. I keep on track with them (p. 313) Rief et al. (2017)	U
Virtual relationships	It felt like she cared and there was a relationship there, er… and I trusted her and respected what she said, and her knowledge and experience (Participant W, p.6) Atkinson (2023)	U
Feeling confident being face‐to‐face on the VC	It felt like now she is coming into our home in a picture it was only because it was something new that it felt strange but then it was, it was quite … natural (Father, p.362) Lindberg, Christensson, and Ohrling (2009)	U
Interacting with the genetic clinician	I could have said anything to her, like I could have with the normal doctors that I see. I didn't get the feeling I was any less entitled to be there or inferior or anything. I found her to be very professional in the way she spoke and the way she handled herself. I felt very well treated and privileged, I suppose, to have that time (018, p. 468) Zilliacus et al. (2010)	C
Interacting with the genetic counsellor	I think [the genetic counsellor] let me know that she was there as a backup support because she knew a lot of the family history and when [the genetic clinician] asked a question, [the genetic counsellor] was there to say—if I didn't know [the genetic counsellor] would answer for me (013, p. 468) Zilliacus et al. (2010)	C
Category 7: Competence in listening to the patient		
Patient perspective	Dr […] got right to the point and solved my problem. I get anxious before talking to doctors and most authority figures, in general. Seeing someone listen, understand, and smile back at me is a huge relief. I appreciate that she did those things. I feel better already (p. 311) Elliott et al. (2020)	C
Listens, is attentive	Great patience in dealing with my son and me. Actually listens to what you're saying (p. 311) Elliott et al. (2020)	U
Emotional contact	I felt that everything was very impersonal and at times a little unprofessional. I missed some acknowledgement of my feelings and ‘problems’. Instead, it turned out to be a mechanical follow‐up with focus on finishing the programme and deadlines (p.4) Sayar, Vøllestad, and Nordgreen (2023)	U
Social dimension: from diffuse to specific relationships	That human interaction to me is important, that empathy, that understanding … the consultant that I see has seen me through quite a lot of difficult times, he knows the history … it was very helpful for me, if I came in with an issue … he sort of understood where I was coming from and I didn't have to sort of explain it all over again (P104_Br_i, p.1129) Marent et al. (2021)	U
Category 8: Competence in creating a comfortable atmosphere		
Social presence	No I didn't find it impersonal or anything. I felt like [the genetic clinician] was still more or less—I felt that she was very close actually. I didn't feel that she was miles away from you. It made me feel comfortable about it all (014, p. 468) Zilliacus et al. (2010)	U
Builds rapport	She was extremely helpful, professional and knowledgeable of my situation. But the reason it was an awesome experience, she made me feel as if she cared about my concerns as a patient by detailing every question asked, creating an atmosphere of comfortability (p. 311) Elliott et al. (2020)	U
Lack of practitioners' counselling skills	… But when they go online, they turn out to be like, awkward… (Patient 06, p.8) Roslan et al. (2024)	C
Expectation and agenda setting	He made me feel comfortable by telling me what he was doing while talking to me and why he was doing it (p. 311) Elliott et al. (2020)	C
Spent right amount of time	Dr […] was extremely friendly, professional, and took the time to understand my symptoms and never made me feel rushed! (p. 312) Elliott et al. (2020)	U
Physical environment	Explain to everybody you know, … don't have somebody walking in and out of the room while you're on Zoom (G05, p.7) Kringle et al. (2023)	U
Experience using telemedicine	[The nurse] was very personable and very straightforward and kind of funny, and really kind of put me at ease through the whole thing (ID 12, 217 miles, surgical abortion, p.411) Ehrenreich et al. (2019)	C

**TABLE 5:** Data synthesis of findings into categories and synthesis findings.

Findings (*n* = 41)	Categories (*n* = 8)	Synthesised findings (*n* = 3)
Zoom functions Checklist Demonstration Repetition Written materials Contrasting experiences with using technology	Competence in providing digital support to patients	1. Competence to provide efficient digital counselling Patients experienced that HCPs need competence in providing digital support to them, in addition to solving technological problems
Technology disruptions Collective action	2 Competence in solving technological problems	
Elicits information Shares decision‐making Provides treatment	3 Competence in making treatment‐related decisions	2. Competence to support patient self‐management during digital counselling Patients felt it was important for HCPs to be competent in informing patients about their disease and its treatment, making treatment‐related decisions and providing instructions related to self‐management
Written asynchronous communication offers an accessible way to seek help after early discharge Shares information/provides guidance Pushing Tailored contact Personalised care meeting and supporting individual needs Feelings of connectedness with peers and professionals Reflexive monitoring Patient‐driven communication	4 Competence in providing instructions related to self‐management	
Virtual consultation empowered self‐advocacy Counselling on how to live with endometriosis Comprehensive support for self‐care Counselling on endometriosis and its role in life Improved physical and mental health Knowing your disease	5 Competence in providing patients with information about their disease and its treatment	
Partnering with providers Virtual relationships Feeling confident being face‐to‐face on the VC Interacting with the genetic clinician Interacting with the genetic counsellor	6 Competence in building a trusting relationship	3. Competence in establishing a reciprocal relationship in digital counselling HCPs need competence in creating a comfortable atmosphere during digital counselling, as well as building a trusting relationship with patients. Moreover, patients described competence in listening to patients as being important
Patient perspective Listens, is attentive Emotional contact Social dimension: from diffuse to specific relationships	7 Competence in listening to the patient	
Social presence Builds rapport Lack of practitioners' counselling skills Expectation and agenda setting Spent right amount of time Physical environment Experience using telemedicine	8 Competence in creating a comfortable atmosphere	

#### 4.3.1 Synthesised Finding 1: Competence to Provide Efficient Digital Counselling

The first synthesised finding included two categories which reflected eight findings. The first category, competence in providing digital support to patients, was supported by six findings. It was important to patients that the HCP was comfortable in using the digital tools concerned (Kringle et al. 2023). Patients found it helpful when the HCP knew how to share their screen to show the patient where to click and what to look at on their device (Kringle et al. 2023). In fact, providing digital support to the patient was seen as a crucial facilitator of digital counselling. Patients suggested that an HCP should provide digital support, for example, by organising a training session on the use of the relevant technology (O'Brien et al. 2023), creating a checklist of the technological functions that the patient should know how to use (Kringle et al. 2023), and giving patients written instructions and time to experiment with the technology before using it in a counselling session (Kringle et al. 2023).

Two findings supported the synthesised category of competence in solving technological problems. A participant in one study (Kringle et al. 2023) mentioned that the HCP was efficient and solved a problem without significant distractions. In the case of connection challenges, HCPs would solve the problem by giving the patient a call or changing to a more functional software option (Gilbert et al. 2022).

#### 4.3.2 Synthesised Finding 2: Competence to Support Patient Self‐Management During Digital Counselling

The second synthesised finding included three categories which reflected 17 findings. The category competence in making treatment‐related decisions was supported by three findings. HCPs needed the skills to ensure that both the facts and the patient's concerns were considered in decision‐making. Patients also appreciated being involved in planning their treatment (Elliott et al. 2020). Patients felt that it was important that HCPs elicit information from them, for example, asking questions about their current health status (Elliott et al. 2020), and provide treatment correctly, for example, prescribing the appropriate medication for a sinus infection (Elliott et al. 2020).

The category competence in providing instructions related to self‐management was supported by eight findings. Patients mentioned that, in addition to professional skills, it is beneficial if HCPs have the communication skills (O'Brien et al. 2023) to push and challenge them to engage in the treatment (O'Brien et al. 2023; Sayar, Vøllestad, and Nordgreen 2023). They also felt that personalised care which reflects their individual needs is essential in digital counselling (O'Brien et al. 2023), and they appreciated HCPs who listened carefully and provided detailed instructions (Elliott et al. 2020). Tailored advice, such as concrete and specific feedback on likely obstacles and challenges, was also considered to be important (Sayar, Vøllestad, and Nordgreen 2023). Text‐based counselling enables patient‐driven communication when patients have questions about their conditions (Rief et al. 2017) and offers an accessible way of seeking help. In this context, patients felt it was important that HCPs write clear and understandable instructions with adequate justification (Danbjørg et al. 2015). On the other hand, reflexive monitoring, for example, correcting patients' movements via video connection, was perceived to be inaccurate, particularly if video quality was not good (Gilbert et al. 2022).

The category competence in providing patients with information about their disease and its treatment was supported by six findings. Patients felt that digital counselling improved their physical and mental health. Nevertheless, they wished that HCPs would provide more information about physical changes associated with their disease (O'Brien et al. 2023). Patients described that it was essential for HCPs to give comprehensive support for self‐care, and thus vital that HCPs had expertise in their disease (Remes, Hakala, and Oikarinen 2023). Counselling about a disease, its role in the patient's life (Remes, Hakala, and Oikarinen 2023) and how to live with it, including supporting loved ones, (Remes, Hakala, and Oikarinen 2023) were perceived as important aspects of digital counselling. Patients described that digital counselling helped them to better understand their disease if they were given advice about treating and monitoring it (Nissen and Lindhardt 2017). Digital counselling empowered their self‐advocacy, for example, when HCPs motivated patients to test their blood pressure and then discussed its health significance with them (Dubrofsky et al. 2023).

#### 4.3.3 Synthesised Finding 3: Competence in Establishing a Reciprocal Counselling Relationship in Digital Counselling

The third synthesised finding included three categories which reflected 16 findings. The category, competence in building trusting relationship, was supported by five findings. According to Rief et al. (2017), patients were partners with providers, that is, patients were responsible for their own care, but they expected HCPs to provide counselling on current issues. Patients experienced that virtual relationships required trust and respect, and building this was influenced by the HCP's knowledge and experience (Atkinson 2023). In addition, professionalism created trust with patients and was reflected in the way that HCPs spoke and behaved during digital counselling (Zilliacus et al. 2010). For example, video‐mediated counselling felt natural when the HCPs knew that facing the patient would invite their trust (Lindberg, Christensson, and Ohrling 2009). Knowing the patient's history beforehand also increased the sense of trust during digital counselling (Zilliacus et al. 2010).

Competence in listening to the patient was supported by four findings. It was important that HCPs consider patients' perspective when solving their problems. For example, listening, understanding and smiling back could help ease a patient's distress (Elliott et al. 2020). If the focus is limited to solving a problem, digital counselling can feel emotionally detached (Sayar, Vøllestad, and Nordgreen 2023), while attentive listening or expressions of patience facilitate interaction (Elliott et al. 2020). When an HCP knew a patient's specific history this helped them to understand the patient's situation and led to more empathic interaction during the counselling (Marent et al. 2021).

The category competence in creating a comfortable atmosphere included seven findings. It was considered essential that HCPs were socially present (Zilliacus et al. 2010). Further, patients felt that building rapport contributed to a comfortable atmosphere and could be achieved by focusing on their concerns and answering their questions in detail (Elliott et al. 2020), involving the patient in setting expectations and the agenda (Elliott et al. 2020), and spending the right amount of time with them, without rushing (Elliott et al. 2020). HCPs' experience in telemedicine enabled them to interact in personal and straightforward ways, helping the patient to participate in digital counselling (Ehrenreich et al. 2019). On the other hand, if the HCP did not have sufficient counselling skills, the digital counselling situation could be awkward (Roslan et al. 2024). Patients felt also that HCPs needed the competence to create an undisturbed physical environment for video‐mediated counselling (Kringle et al. 2023).

## 5 Discussion

This systematic review and qualitative meta‐aggregation aimed to synthesise patients' experiences of HCPs' competence in digital counselling in healthcare settings. Three synthesised findings were identified based on 42 findings and eight categories. The first interesting finding concerned digital competence, which is a prerequisite for healthcare, including counselling, to be implemented digitally (Leonardsen et al. 2020). It could be argued that, without fluent digital competence on both the professional's and the patient's side, the central purpose of counselling cannot be achieved (Kaihlanen et al. 2023). This finding relates to both the HCP's own digital competence and their ability to solve problems arising for the patient during the counselling situation. Similar results have also been found from the perspectives of healthcare professionals (Konttila et al. 2019; Kaihlaniemi et al. 2023; Jarva et al. 2022). Digitalisation has created new areas of competence for HCPs, such as providing digital support to patients, and this was also reflected in the results of this study. HCPs need accessible, valid tools for assessing patients' prerequisites for digital counselling to identify support needs and remove barriers rather than exclude anyone from digital services (Kaihlanen et al. 2023).

The second finding, competence to support patient self‐management during digital counselling, is an area that represents the fundamental competence of healthcare practice and is particularly crucial in effective counselling. It demands that healthcare professionals apply their skills flexibly, employing diverse approaches to help patients adhere to their own care, as shown by earlier studies (Oikarinen et al. 2018; Kähkönen et al. 2023). However, in contrast to the past, counselling is now more often implemented digitally (Härkönen et al. 2024). Our results emphasise the importance of HCPs being able to apply diverse and adaptable knowledge during counselling, for example, understanding the patient's disease and treatment, as previous studies have also concluded that providing personalised information and advice is essential to increasing patients' ability to self‐manage their condition (Oikarinen et al. 2018; Beishuizen et al. 2019). In line with previous studies (Kaakinen et al. 2020; Kaihlaniemi et al. 2023; Oikarinen et al. 2018), our results suggest that competence in providing clear instructions relating to self‐management is essential to counselling. Feelings of connectedness between patient and professional are enabled when information is shared in a patient‐driven way and the patient's situation and motivation are evaluated using participatory approaches. Patients also expect HCPs to have the competence to make treatment‐related decisions in collaboration with the patient in the digital counselling environment, as earlier studies have also argued (Leonardsen et al. 2020).

The third finding, competence in establishing a reciprocal counselling relationship during digital counselling, is crucial to ensuring continuity of holistic care. A previous systematic review found that digital interaction between HCPs and patients with long‐term conditions promoted continuity of care (Hopstaken et al. 2021). The study suggests that patients anticipate HCPs to be skilled in building trusting relationships, an area where previous research has indicated some challenges (Gordon et al. 2020). Therefore, the means of sufficient communication in digital counselling should be considered in curricula and in healthcare education. Such competence is more likely to arise when the HCP is someone familiar to the patient, who communicates naturally and is experienced in the subject. The more knowledge and experience an HCP had, the more patients felt they could trust them. Our results also emphasised that listening to the patient promoted reciprocity. Therefore, understanding the patient's situation and empathic encounters were crucial but, based on the experience of professionals, this is challenging (Kaihlaniemi et al. 2023; Laukka et al. 2020). It should be recognised that, if the focus in digital counselling is solely on solving problems, the situation can become emotionally distant. Thus, digital emotional intelligence, which has been described as the integration between emotional intelligence and digital competence (Audrin and Audrin 2023), is essential. Our results described that the HCP must know how to create a comfortable environment for digital counselling. This was enhanced by features such as understanding the patient's symptoms, meeting the patient's expectations, setting a timeframe and ensuring that the counselling situation was not rushed. The HCP's experience of telemedicine also helped the patient to participate in digital counselling. However, disruption in the physical environment prevented the creation of a comfortable atmosphere. It should be noted that the HCP's counselling skills are important in preventing the counselling situation from becoming uncomfortable from the patient's point of view.

Current digital health frameworks concentrate on the development of HCPs' digital skills, proficiency in managing health‐related information and digital communications and awareness of ethical, legal, privacy and security implications relating to digital services (Nazeha et al. 2020). In this study, privacy and security skills were emphasised in the context of creating a calm environment during video‐mediated counselling. Other information security‐related findings did not emerge, in contrast to other studies into digital counselling competencies for professionals (Kaihlaniemi et al. 2023; Jarva et al. 2024).

Further, it seems that the same issues apply to digital counselling as apply to traditional, face‐to‐face counselling. Traditionally, good counselling has been described as interactive, patient‐centred, carefully planned and implemented with sufficient resources (Kaakinen et al. 2020; Oikarinen et al. 2018, 2023). Interaction is fundamental because achieving the objectives of counselling, in terms of patient action and self‐care, relies on establishing a successful interactive relationship.

## 6 Conclusion

This systematic review establishes an evidence base, from the perspective of patients, which can be used to develop digital counselling services in healthcare settings and build the competence of HCPs. From patients' experiences, it was identified that HCPs' digital counselling competence is a multidimensional entity which relates to many other core competencies within their work. Specifically, it draws on digital competence, competence in supporting patients' self‐management and competence in establishing reciprocal counselling relationships in a digital environment.

The research provides a basis for improving HCPs' proficiency in digital counselling, ultimately fostering a more patient‐centred approach to care. By integrating various elements of digital counselling, HCPs can greatly influence patients' long‐term health outcomes across different healthcare environments. In the education and training of HCPs, it is essential to cover all facets of digital counselling competence to ensure they develop the highest level of expertise, ultimately benefiting patient health.

## 7 Limitations

While the study was meticulously designed to ensure thoroughness and rigour, it is essential to acknowledge and address certain limitations. Firstly, despite following the JBI guidelines for systematic reviews (Lockwood et al. 2020) and PRISMA reporting guidelines (Page et al. 2021), including an information specialist in the study team, and consulting numerous databases with a range of keywords, it is possible that some studies pertinent to the review topic may have been overlooked. The second challenge pertains to the abstract and intricate nature of concepts such as counselling and competence, which pose challenges to research. The research group aimed to overcome this by providing clear descriptions of the contents at the outset and engaging in ongoing discussions throughout the review process. Over 14,000 original articles were identified through the search strategy, but only 16 studies were selected. Our large and experienced research team conducted several practice searches to ensure the robustness of the systematic search. More specifically, a precise research question and criteria were established, and a systematic search strategy was implemented, resulting in the systematic selection of articles. Further, the search was limited to languages known to the research team, restricting our ability to identify studies reported in other languages. Additionally, because the review focused on qualitative studies, caution is required when generalising its results. Further, most of the studies included were carried out in Western countries, introducing a potential bias that may hinder the generalisability of the results to more diverse countries and cultures.

## Author Contributions

J.K., P.S. and A.O. made substantial contributions to the conception or design of the work or the acquisition, analysis or interpretation of data for the work. J.K., P.S., M.K., P.K., M.L., L.P., K.L. and A.O. were involved in drafting the work or reviewing it critically for important intellectual content. J.K., P.S., M.K., P.K., M.L., L.P., K.L. and A.O. gave final approval of the version to be published. J.K., P.S., M.K., P.K., M.L., L.P., K.L. and A.O. agreed to be accountable for all aspects of the work in ensuring that questions related to the accuracy or integrity of any part of the work are appropriately investigated and resolved.

## Conflicts of Interest

The authors declare no conflicts of interest.

## Peer Review

The peer review history for this article is available at https://www.webofscience.com/api/gateway/wos/peer‐review/10.1111/jan.16663.

## Supporting information


Supplementary File 1

## Data Availability

The authors have nothing to report.
